# Corticobasal syndrome mimicking Foix-Chavany-Marie syndrome with suggested 4-repeat tauopathy by tau PET

**DOI:** 10.1186/s12877-023-04564-z

**Published:** 2023-12-12

**Authors:** Kosei Nakamura, Yasuko Kuroha, Masahiro Hatakeyama, Atsushi Michael Kimura, Yukimi Nakamura, Yoshihiro Murakami, Masaki Watanabe, Hironaka Igarashi, Tetsuya Takahashi, Hitoshi Shimada

**Affiliations:** 1 Department of Neurology, Clinical Neuroscience Branch, Brain Research Institute, Niigata University, Niigata, Japan; 2 Department of Functional Neurology & Neurosurgery, Center for Integrated Human Brain Science, Brain Research Institute, Niigata University, 1-757 Asahimachi-Dohri, Niigata, 951-8585 Japan; 3 Department of Neurology, NHO Nishiniigata Chuo Hospital, Niigata, Japan; 4 Department of Integrated Neuroscience, Center for Integrated Human Brain Science, Brain Research Institute, Niigata University, Niigata, Japan; 5 Department of Biological Magnetic Resonance, Center for Integrated Human Brain Science, Brain Research Institute, Niigata University, Niigata, Japan; 6 Department of Functional Brain Imaging, Institute for Quantum Medical Science, Quantum Life and Medical Science Directorate, National Institutes for Quantum Science and Technology, Chiba, Japan

**Keywords:** Corticobasal syndrome, Foix-Chavany-Marie syndrome, Tau PET, Aphasia

## Abstract

**Background:**

Corticobasal syndrome (CBS) is a neurodegenerative disease diagnosed based on clinical manifestations such as asymmetrical parkinsonism, limb apraxia, and speech and language impairment. The background pathology of CBS is commonly a variety of proteinopathies, but association with cerebrovascular disease has also been reported. Foix-Chavany-Marie syndrome (FCMS) is a rare neurological disorder characterized by facio-pharyngo-glossal diplegia with automatic-voluntary movement dissociation presenting with bilateral paresis of the facial, lingual, pharyngeal and masticatory muscles. FCMS is commonly attributable to stroke. Transactive response DNA binding protein of 43 kD (TDP-43) proteinopathy is also known as the pathological background of FCMS, while the pathological background of the majority of CBS cases consists of diverse tauopathies instead of TDP-43 proteinopathy. In this report, we describe a case mimicking FCMS that was finally diagnosed as CBS with suggested 4-repeat tauopathy.

**Case presentation:**

A 68-year-old female started experiencing difficulty speaking followed by difficulty writing, and especially texting, several years before her visit. Her impairment had been gradually worsening, and she came to our hospital. On neurological examination, she demonstrated the facial apraxia, frontal lobe dysfunction, and upper motor neuron signs. She presented some characteristics suggestive of FCMS. Her symptoms exhibited rapid progression and myoclonus, parkinsonism, and left-side dominant cortical sensory deficit occurred, resulting in the fulfillment of diagnostic criteria for CBS after 9 months. Tau PET imaging displayed notable ligand uptake in the brainstem, subthalamic nuclei, basal ganglia, and bilateral subcortical frontal lobe, suggesting that her pathological background was 4-repeat tauopathy. As a result of her progressive dysphagia, she became unable to eat and passed away after 12 months.

**Conclusion:**

We hereby present an atypical case of CBS showing clinical features mimicking FCMS at first presentation. TDP-43 proteinopathy was suspected based on the clinical symptoms in the early stages of the disease; however, the clinical course and imaging findings including tau PET suggested that her pathological background was 4-repeat tauopathy.

## Background

Corticobasal syndrome (CBS) is a neurological disease diagnosed based on clinical manifestations such as asymmetrical parkinsonism, limb apraxia, and speech and language impairment. The heterogeneous underlying pathology of CBS encompasses 4-repeat tauopathies, notably corticobasal degeneration (CBD) and progressive supranuclear palsy (PSP), alongside cerebrovascular afflictions, which may also serve as etiological factors [1]. Foix-Chavany-Marie syndrome (FCMS), also known as anterior opercular syndrome, is a rare neurological disorder characterized by facio-pharyngo-glossal diplegia with automatic-voluntary movement dissociation presenting with bilateral paresis of the facial, lingual, pharyngeal and masticatory muscles. While stroke is the predominant cause of FCMS, it can also be induced by neurodegenerative disorders [2]. Several reports on FCMS have described transactive response DNA binding protein of 43 kD (TDP-43) proteinopathy as the background pathology [3, 4]. Here, we report a case of CBS presenting with clinical features mimicking FCMS at the early stage of the disease. In this context, tau positron emission tomography (PET), in addition to the clinical course and other imaging findings, was considered useful for the diagnosis.

## Case presentation

**Fig. 1:**
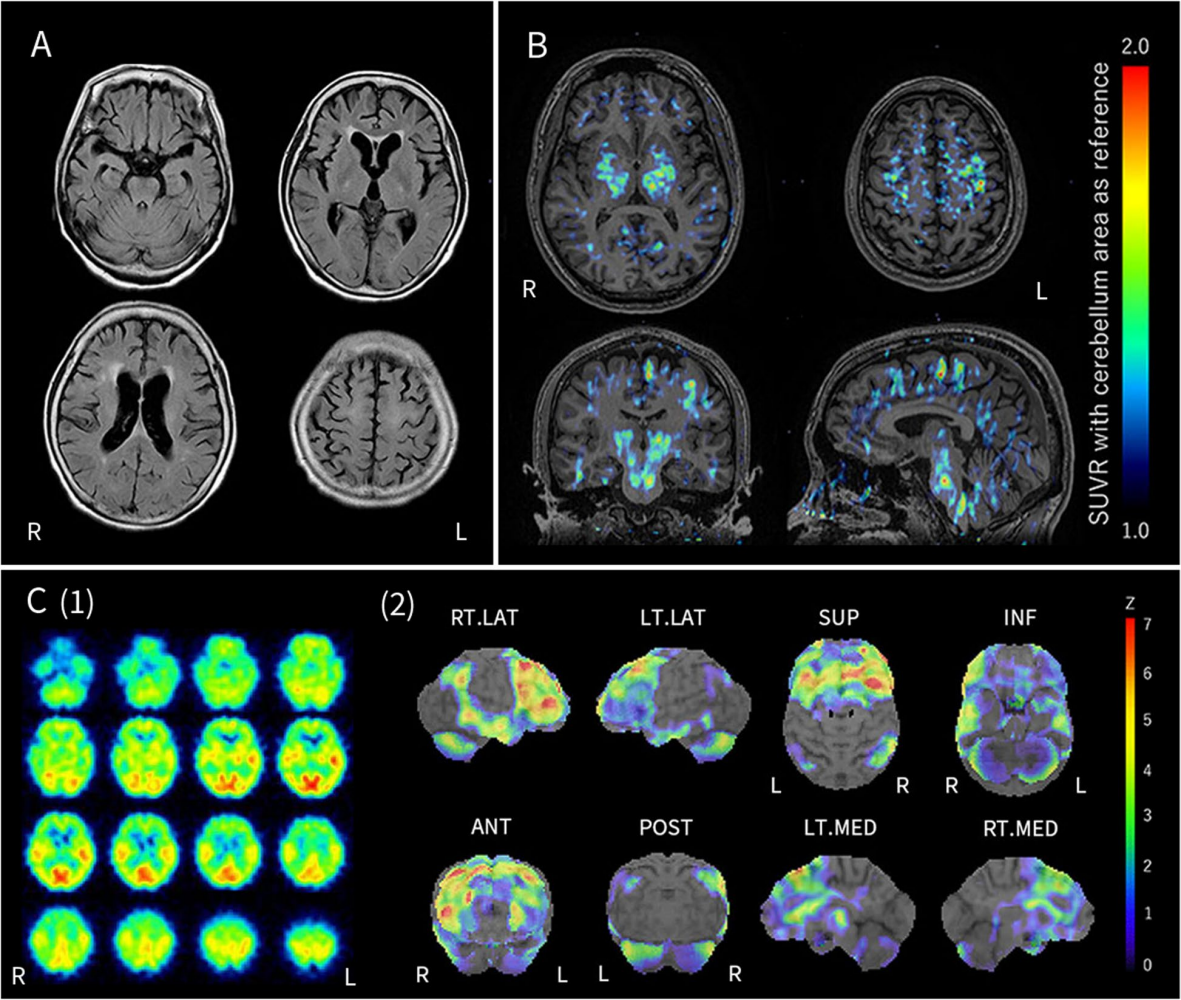
MRI, tau PET, and perfusion SPECT images. Brain magnetic resonance imaging revealed bilateral frontotemporal lobar atrophy on T2/fluid-attenuated inversion recovery (FLAIR) (**A**). Tau positron emission tomography (PET) with Florzolotau (^18^F) demonstrated remarkable uptake of the ligand around the brainstem, subthalamic nucleus, basal ganglia, and bilateral subcortical frontal lobe (**B**). ^123^I-N-isopropyl-p-iodoamphetamine (^123^I-IMP) single-photon emission computed tomography (SPECT) showed reduced cerebral blood flow in the bilateral frontal lobe and basal ganglia (C-1). Easy Z-score imaging system (eZIS) analysis of ^123^I-IMP SPECT imaging also revealed significantly decreased regional cerebral blood flow (rCBF) in bilateral frontal areas, including the anterior operculum (C-2). SUVR, standardized uptake value ratio; RT, right; LT, left; LAT, lateral; SUP, superior; INF, inferior; ANT, anterior; POST, posterior; MED, medial

A 68-year-old right-handed woman, who had a 12-year education, had difficulty speaking for the last several years. She was treated for hypertension but had no hyperlipidemia, no diabetes, and no history of smoking. Approximately seven months ago, she started making kana-dominant spelling mistakes specifically in her mobile text messages. Of note was that the frequency of errors in her mobile text messages exceeded those observed in her handwriting, suggesting dystextia. When she visited our hospital, she exhibited apraxia of speech, eyelid opening, oculomotor, and buccofacial movement. There was dissociation between spontaneous and voluntary movements of facial expression. Perseveration, pathological laughing, and increased limb tendon reflexes were prominent. Lower motor neuron signs were absent from all extremities and her tongue. Her Mini-Mental State Examination (MMSE) score was 19/30 and her Frontal Assessment Battery (FAB) score was 7/18, suggesting mild cognitive decline including frontal lobe dysfunction. Detailed neuropsychological evaluation by Western Aphasia Battery (WAB) for Japanese (shortened version) is shown in Table 1. Based on the observed irregular stagnation and distortion in her spontaneous speech, it appeared that she was suffering from apraxia of speech rather than dysarthria. Furthermore, although her comprehension of complex sentences was impaired, single-word comprehension remained intact. Brain magnetic resonance imaging (MRI) and perfusion single photon emission computed tomography (SPECT) showed bilateral atrophy and decreased cerebral blood flow in the frontotemporal lobe, including the anterior operculum (Fig. 1A and C). Visual assessment of the dopamine transporter (DaT) scan revealed normal symmetrical uptake in the caudate and putamen region. The specific binding ratio (SBR) calculated as the ratio of striatal specific to non-specific binding was 5.27 on the right and 5.29 on the left, within normal range for this same age group. Cervical MRI failed to show any lesions that might explain the increased tendon reflexes. Needle electromyography showed no obvious abnormalities suggestive of motor neuron diseases. After a while she also presented with dysphagia. In this case, bilateral paresis of the facial, lingual, pharyngeal and masticatory muscles was not observed, so we cannot exactly label it FCMS, but the patient had paralytic-like symptoms due to apraxia, which resembled FCMS.

**Table 1:**
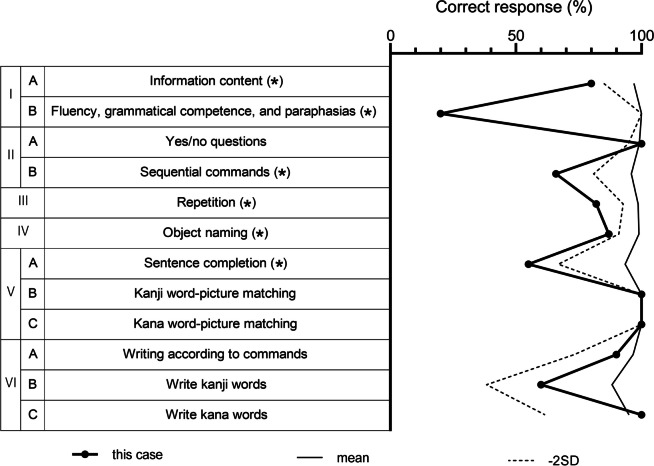
Result of Western Aphasia Battery for Japanese (shortened version)

I: Spontaneous speech, II: Auditory verbal comprehension, III: Repetition, IV: Object naming, V: Reading, VI: Writing. Asterisks denote items with mean – 2SD. Mean and – 2SD for each of the lower test items in healthy subjects were as follows: I—A: 97.0 ± 12.0%, I—B: 100%, II—A: 99.2 ± 4.4%, II—B: 96.0 ± 15.0%, III: 98.7 ± 6.0%, IV: 99.0 ± 8.0%,V—A: 93.5 ± 26.6%, V—B: 100%, V—C: 100%, VI—A: 96.7 ± 23.4%, VI—B: 88.3 ± 50.0%, VI—C: 95.0 ± 33.4%. SD, standard deviation

**Fig. 2:**
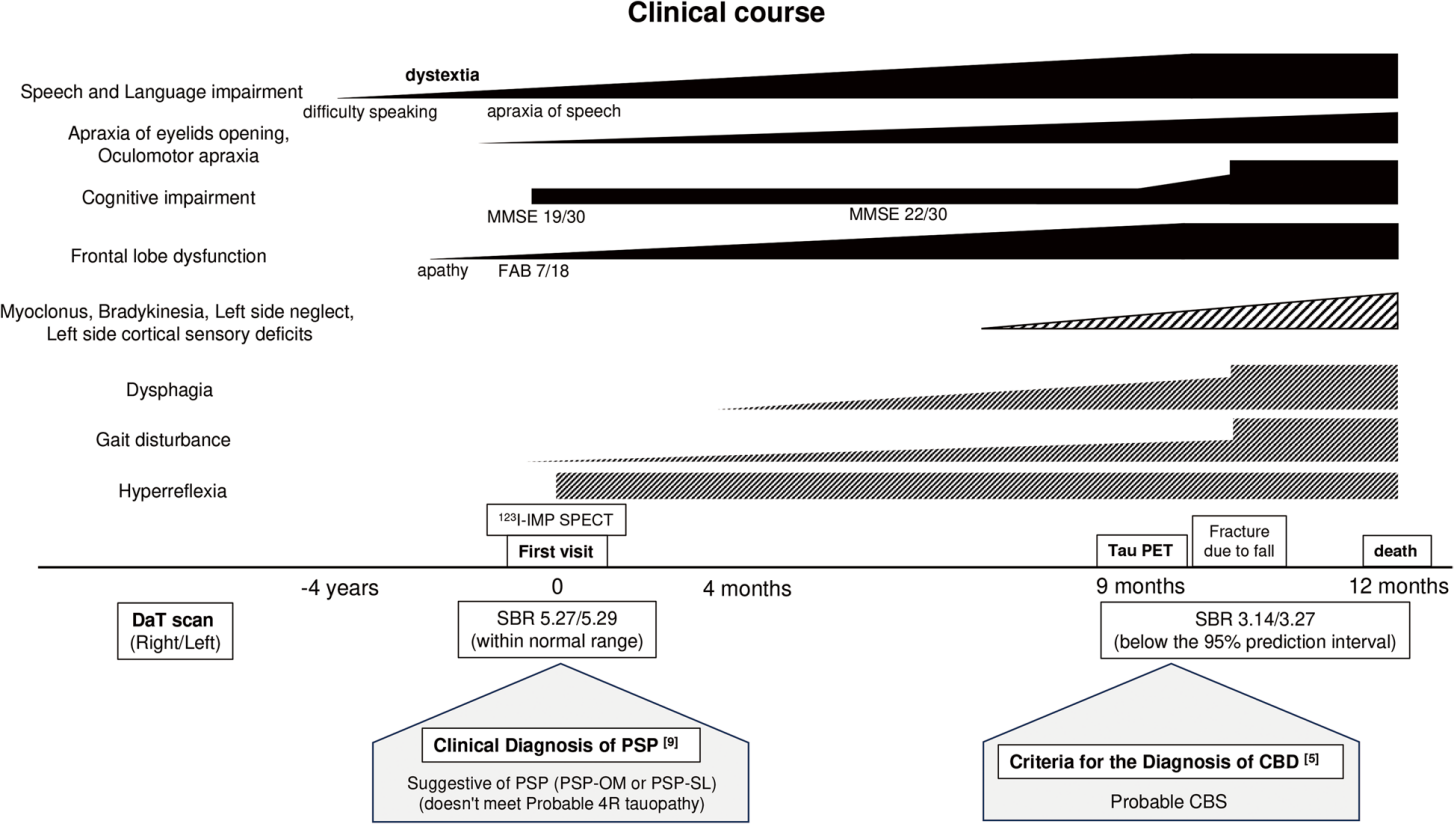
Clinical course of this case. The clinical course of the present case is shown. She had difficulty speaking, subsequently exhibiting dystextia and apraxia of speech. Initial evaluation did not reveal indications of probable 4R tauopathy. However, with disease progression, the emergence of myoclonus, bradykinesia, left-sided neglect, and cortical sensory deficits on the left side occurred. Consequently, her criteria pointed to probable CBS and raised suspicions of underlying 4R-tauopathy. MMSE, Mini-Mental State Examination; FAB, Frontal Assessment Battery; SPECT, Single Photon Emission Computed Tomography; DaT, Dopamine Transporter; SBR, Specific Binding Ratio; PSP, Progressive Supranuclear Palsy; PSP-OM, PSP with predominant ocular motor dysfunction; PSP-SL, PSP with predominant speech/language disorder; CBD, Corticobasal Degeneration; CBS, Corticobasal Syndrome

Her clinical symptoms continued to gradually worsen, and she presented with myoclonus, bradykinesia, postural instability, left-side neglect, and left-side dominant cortical sensory deficit nine months later (Fig. 2). Tau PET with Florzolotau (^18^F) demonstrated remarkable uptake of the ligand around the brainstem, subthalamic nucleus, basal ganglia, and bilateral subcortical frontal lobe (Fig. 1B), while amyloid PET with ^11^C-PiB was negative by visual assessment. Based on neurological findings, she was ultimately diagnosed with probable CBS [5], and the tau PET image suggested that her pathological background was CBS-4repeat-tauopathy [6]. Ten months after her first diagnosis, she was hospitalized for a tibia fracture due to a fall. With the progression of her symptoms, she exhibited severe dysphagia and communication difficulties, eventually becoming bedridden. After conducting a follow-up DaT scan, a diffuse decrease in bilateral striatal uptake was observed, with SBR measuring 3.14 on the right and 3.37 on the left, both below the 95% prediction interval for the same age group. As a result of the progressive dysphagia, she became unable to eat and passed away 12 months after her first diagnosis. Unfortunately, consent for an autopsy could not be obtained.

## Discussion

In this case, the initial symptom was her speech difficulty, which was followed by dystextia. Dystextia involves the phenomenon of the inability to compose and send text messages. The Japanese written language consists of both kana (syllabograms) and kanji (ideograms). Previous studies indicated that kana and kanji processings may occur in distinct brain regions, as evidenced by the occasional impairment of kana selectivity in cases of frontal or parietal lesions in Japanese amyotrophic lateral sclerosis (ALS) patients, being the predominant motor neuron disease characterized by the accumulation of TDP-43 [7]. When constructing sentences in Japanese mobile text messages, individuals only need to select kana characters displayed on the screen and press the corresponding buttons, as opposed to handwriting for which they must recall the letter configurations. In instances of kana impairment, it is possible that the frequency of errors could be greater during text input compared to handwriting in Japanese ALS patients [8]. While no case of CBS presenting with dystextia has been reported thus far, it may actually be considered an early manifestation of CBS, as in the present case.

When the patient was initially examined, she exhibited hyperreflexia and dystextia, displaying some features of FCMS. Although there are few autopsy reports of FCMS, TDP-43 is known as the background pathology. FCMS is often seen in bilateral anterior operculum lesions by brain MRI. In this case, MRI showed bilateral atrophy of the anterior operculum, and perfusion SPECT showed decreased blood flow in certain regions including the bilateral anterior operculum. Based on the clinical findings at her first visit, PSP with predominant speech/language disorder (PSP-SL) was also considered; however, she did not meet probable 4-repeat tauopathy by PSP criteria [9], and DaT scan showed no evidence of decreased uptake in the bilateral striatum. Hence, we postulated that she was in the preliminary stage of motor neuron disease and would exhibit muscular weakness in the imminent timeframe. Nonetheless, upon subsequent observations, she did not exhibit the typical progression of motor neuron disease. Instead, she met the diagnostic criteria for probable CBS, and the tau PET with Florzolotau (^18^F) findings suggested 4-repeat tauopathy. Florzolotau (^18^F) sometimes shows off-target binding to the choroid plexus, but no cross-reactivity to monoamine oxidase -A/-B, resulting in high binding affinity and specificity for evaluating tau pathology in the brain parenchyma [6]. Occasionally, degeneration of the corticospinal tract has been reported in 4-repeat tauopathies such as CBD and PSP [10]. Since it is challenging to distinguish pyramidal slowness from bradykinesia and spasticity from rigidity [11], upper motor neuron signs may be more prevalent in 4-repeat tauopathies than previously assumed.

Our case lacked the obvious asymmetry in imaging findings known as classically characteristic features observed in non-fluent/agrammatic variant Primary Progressive Aphasia (nfvPPA); however, she met the clinical diagnostic criteria for nfvPPA, especially based on the results of neuropsychological testing [12]. NfvPPA is frequently linked with tauopathy [13], whereas the underlying pathology of FCMS is generally associated with TDP-43. In the present case, a conclusive diagnosis of probable CBS was established, and the tau PET results indicated the presence of 4-repeat tauopathies, such as CBD and PSP. When brain stem atrophy was evaluated using brain MRI, the Magnetic Resonance Parkinsonism Index (MRPI) 2.0 was 2.21 (≥ 2.18) [14]. Perfusion SPECT revealed hypoperfusion in the bilateral frontal lobes and basal ganglia, while DaT imaging demonstrated diffuse hypointensity in the bilateral striatum at the advanced stage of the disease, exhibiting a slight right-dominant pattern consistent with the observed symptoms. These imaging findings also provided grounds for suspecting 4-repeat tauopathy. Although pathological findings are necessary for a diagnosis, globular glial tauopathy (GGT) was also considered in this case. GGT is a relatively recently described 4-repeat tauopathy with a variable clinical presentation that includes motor neuron disease and frontotemporal dementia. Limited studies have reported tau PET findings in GGT [15, 16]; nevertheless, the usefulness of tau PET for the antemortem diagnosis of GGT remains undecided.

MRI findings in CBD may include high signal intensity in subcortical white matter on diffusion weighted image (DWI) and fluid-attenuated inversion recovery (FLAIR), reflecting pathological changes in subcortical white matter [17]. Furthermore, it is important to consider cerebrovascular disease when attempting to determine the cause of FCMS and CBS, since cerebrovascular disease can cause FCMS and CBS [1], and some reports have suggested that vascular pathology may be involved in the development of CBS [18]. In the present case, FLAIR showed only faint high signal intensity lesions in the subcortical region of the frontal lobe and no prominent white matter lesions. Thus, while vascular pathology may be associated with the development of CBS, it is unlikely that cerebrovascular disease itself causes CBS in the present case.

The major limitation of this case was that no autopsy was performed and neuropathological examination results are lacking. The pathology of this case could have been CBD, GGT, PSP, or fronto-temporal dementia.

## Conclusion

In summary, we present an atypical case of CBS presenting with clinical features resembling FCMS at the early stage of the disease. Despite the fact that initial symptoms were similar to the motor neuron disease, she eventually met the diagnostic criteria of probable CBS, with imaging findings including tau PET imaging supporting the presence of 4-repeat tauopathy as the background pathology. As CBS is a heterogeneous syndrome with various clinical presentations, it will be essential to accumulate more cases in order to understand this disease.

## Data Availability

Anonymized raw data supporting the findings of the present study may be shared by the corresponding author upon reasonable request.

## Abbreviations

CBS: Corticobasal Syndrome
CBD: Corticobasal Degeneration
PSP: Progressive Supranuclear Palsy
FCMS: Foix-Chavany-Marie syndrome
TDP-43: Transactive response DNA binding protein of 43 kD
PET: Positron Emission Tomography
MMSE: Mini-Mental State Examination
FDB: Frontal Assessment Battery
WAB: Western Aphasia Battery
MRI: Magnetic Resonance Imaging
DaT: Dopamine Transporter
SBR: Specific Binding Ratio
ALS: Amyotrophic Lateral Sclerosis
PSP-SL: PSP with predominant speech/language disorder
PPA: Primary Progressive Aphasia
nfvPPA: Non-fluent/agrammatic variant PPA
MRPI: Magnetic Resonance Parkinsonism Index
GGT: Globular Glial Tauopathy
DWI: Diffusion Weighted Image
FLAIR: Fluid-Attenuated Inversion Recovery
